# Subgroup Analysis of Pooled Phase 3 Efficacy Data From Two Randomized Trials of Gepotidacin Versus Nitrofurantoin in Uncomplicated Urinary Tract Infections (EAGLE-2 and EAGLE-3) Including Participants With Uropathogens Not-Susceptible to Nitrofurantoin

**DOI:** 10.1093/cid/ciag408

**Published:** 2026-07-08

**Authors:** Amanda Sheets, Jeremy Dennison, Jennifer H Han, Sarah Watts, Caroline Perry, Nicole E Scangarella-Oman, Deborah Butler, John Breton, Thomas M Hooton, Helen Millns, Matthew Helgeson, Salim Janmohamed

**Affiliations:** Vaccine & ID Research Unit, GSK, Collegeville, Pennsylvania, USA; Respiratory, Immunology, and Inflammation Research Unit, GSK, London, United Kingdom; Vaccine & ID Research Unit, GSK, Collegeville, Pennsylvania, USA; Respiratory, Immunology, and Inflammation Research Unit, GSK, London, United Kingdom; Vaccine & ID Research Unit, GSK, Collegeville, Pennsylvania, USA; Vaccine & ID Research Unit, GSK, Collegeville, Pennsylvania, USA; Vaccine & ID Research Unit, GSK, Collegeville, Pennsylvania, USA; Vaccine & ID Research Unit, GSK, Collegeville, Pennsylvania, USA; Infectious Disease Medicine and Internal Medicine, University of Miami, Miami, Florida, USA; Vaccines and Infectious Disease, GSK, Stevenage, United Kingdom; Vaccine & ID Research Unit, GSK, Collegeville, Pennsylvania, USA; Respiratory, Immunology, and Inflammation Research Unit, GSK, London, United Kingdom

**Keywords:** gepotidacin, urinary tract infection, cystitis, antimicrobial resistance

## Abstract

**Background:**

Two phase 3 trials (EAGLE-2 [NCT04020341]/EAGLE-3 [NCT04187144]) demonstrated the efficacy of gepotidacin, a novel, first-in-class triazaacenaphthylene antibacterial, versus nitrofurantoin for the treatment of uncomplicated urinary tract infections (uUTIs). Using pooled data, we assessed gepotidacin efficacy across clinically important subgroups, including participants with nitrofurantoin not-susceptible (NTF-NS; ie, resistant, intermediate or no interpretation) uropathogens.

**Methods:**

Female participants aged ≥12 years with uUTI received gepotidacin (1500 mg) or nitrofurantoin (100 mg), both twice daily for 5 days. Therapeutic success at test-of-cure (day 10–13) was defined as combined complete symptom resolution (clinical success) and reduction of baseline qualifying uropathogens from ≥10^5^ to <10^3^ CFU/mL (microbiological success), without other systemic antibacterials. Response rates at test-of-cure were descriptively summarized in the pooled microbiological intent-to-treat (micro-ITT) nitrofurantoin-susceptible (NTF-S) and NTF-NS populations, and across prespecified subgroups.

**Results:**

In the micro-ITT NTF-S population, treatment differences (in favor of gepotidacin) [95% confidence interval] for therapeutic, clinical and microbiological success were: 9.6% [4.1, 15.2], 2.8% [−2.4, 8.1], and 10.4% [5.2, 15.6]. Corresponding treatment differences were higher in the micro-ITT NTF-NS population: 20.8% [9.2, 32.4], 10.3% [−1.4, 22.1], 20.7% [9.2, 32.3]. In both of these populations, gepotidacin success rates (all endpoints) were similar to or higher than nitrofurantoin across prespecified subgroups at higher risk for antimicrobial resistant uropathogens (age >50 years, history of recurrent uUTI, diabetes, mild renal impairment, extended-spectrum β-lactamase-producing or fluoroquinolone-resistant uropathogens).

**Conclusions:**

Gepotidacin demonstrated efficacy with generally higher therapeutic success rates versus nitrofurantoin in participants with NTF-S and NTF-NS uropathogens, and across a range of key subgroups.

## Graphical Abstract

**Graphical Abstract:**
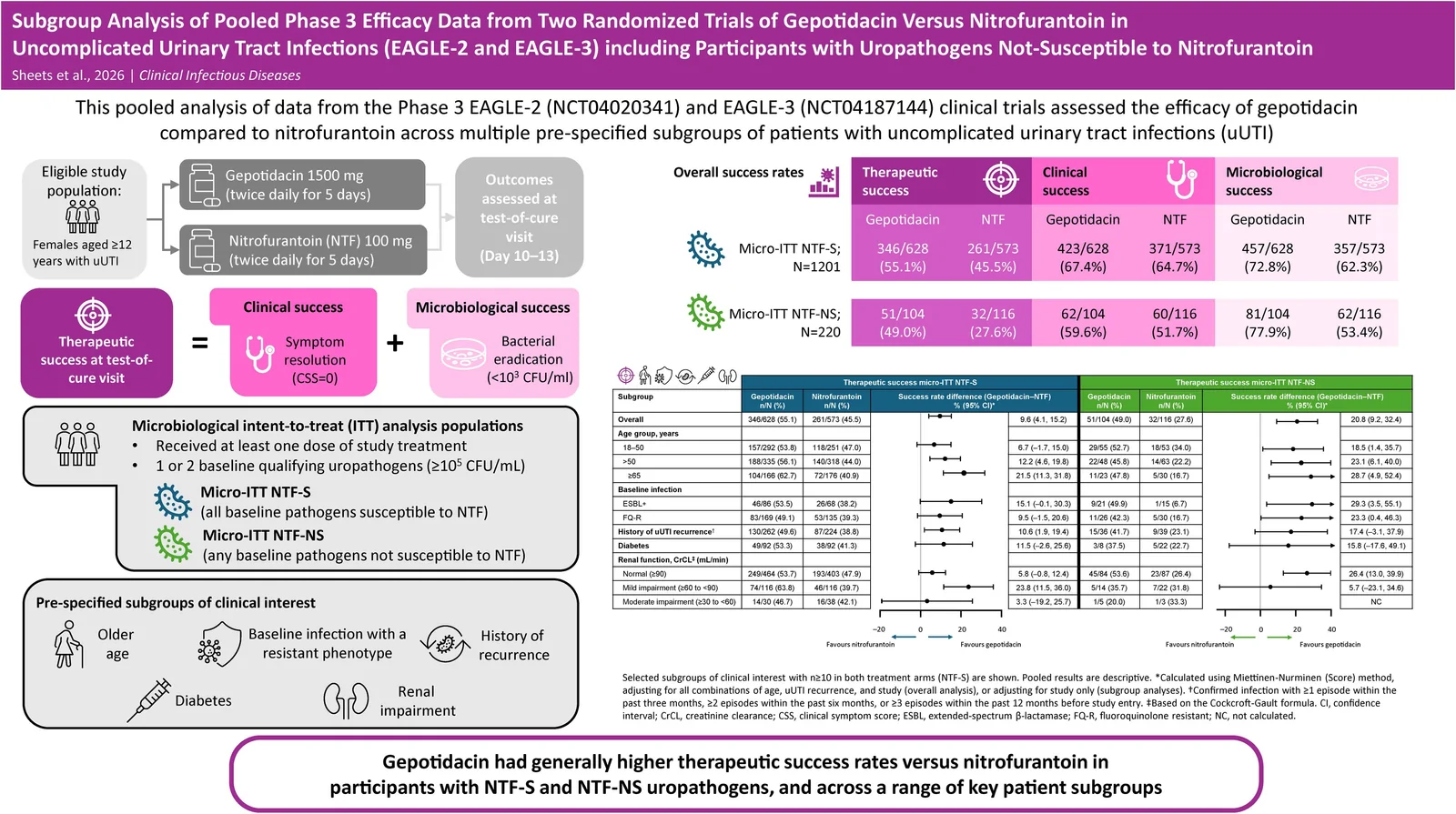

**(See the Editorial Commentary by Geerlings on pages e743–4.)**

Uncomplicated urinary tract infections (uUTIs; acute cystitis) are one of the most common bacterial infections impacting women globally, with a lifetime incidence of 50%–60% [1]. Recurrence rates for uUTIs are high, with approximately 27% of women experiencing a confirmed recurrence within six months of the initial episode [1]. Several demographic and clinical characteristics, including age, history of diabetes and history of recurrent uUTIs, are associated with an increased risk of antibiotic resistance in common uUTI uropathogens and treatment failure [2, 3]. While there are several oral antibacterial drugs available for the treatment of uUTI, their use can be limited by increasing antimicrobial resistance or adverse events. Novel therapeutic agents could help healthcare providers effectively manage uUTIs in patients at risk of treatment failure or for whom current standard of care treatments are unsuitable.

Gepotidacin is a novel, bactericidal, first-in-class triazaacenaphthylene antibacterial that inhibits bacterial DNA replication by a unique mechanism of action and distinct binding site [4, 5]. Gepotidacin provides well-balanced inhibition (for most uUTI uropathogens) of two Type II topoisomerase enzymes (DNA gyrase and topoisomerase IV) rendering it effective against most strains of target uropathogens, including isolates resistant to current antibacterials [6, 7]. Due to the well-balanced binding at both enzymes for most pathogens, a gepotidacin target-specific mutation in a single enzyme does not significantly affect gepotidacin susceptibility [7]. The US Food and Drug Administration (FDA) recently approved gepotidacin for the treatment of female adult and pediatric patients 12 years of age and older weighing at least 40 kilograms (kg) with uUTIs caused by the following susceptible microorganisms: *Escherichia coli*, *Klebsiella pneumoniae*, *Citrobacter freundii* complex, *Staphylococcus saprophyticus*, and *Enterococcus faecalis* [8]. The approval of gepotidacin was based on the results of replicate global phase 3, randomized, controlled clinical trials, EAGLE-2 (NCT04020341) and EAGLE-3 (NCT04187144) [8, 9].

EAGLE-2 and EAGLE-3 demonstrated the efficacy and safety of oral gepotidacin in female participants with uUTI, finding gepotidacin non-inferior to nitrofurantoin in both trials and superior in EAGLE-3 [9]. The current analysis used pooled data from the EAGLE-2 and EAGLE-3 trials to examine outcomes within each treatment arm and to further examine the efficacy of gepotidacin compared with nitrofurantoin across participant subgroups at greater risk of having drug-resistant uropathogens and treatment failure, including those with infections caused by nitrofurantoin not-susceptible (NTF-NS) uropathogens.

## METHODS

### Study Design

EAGLE-2 and EAGLE-3 were phase 3, randomized, multicenter, parallel-group, double-blind, double-dummy, non-inferiority trials in adolescent (12–17 years) and adult female participants comparing oral gepotidacin (1500 mg) to nitrofurantoin (100 mg), both twice daily for 5 days, for the treatment of uUTI. Details regarding the rationale, design, and methods of the EAGLE-2 and EAGLE-3 trials have previously been described [9, 10]. These replicate studies were conducted concurrently without overlapping participating study sites.

The primary endpoint in EAGLE-2 and EAGLE-3 was therapeutic response (success or failure) at test of cure (TOC, day 10–13) assessed in participants with qualifying uropathogens (≥10^5^ CFU/mL) susceptible to nitrofurantoin. Aligned with FDA/European Medicines Agency guidance for registrational uUTI trials, therapeutic success was defined as combined clinical success (complete resolution of symptoms and no new symptoms) and microbiological success (reduction of qualifying bacterial uropathogens from ≥10^5^ CFU/mL at baseline to <10^3^ CFU/mL), without additional systemic antibacterial use [11, 12].

### Study Population

In both trials, eligible participants were non-pregnant biological females, aged ≥12 years, weighing ≥40 kg, and with ≥2 symptoms of uUTI (dysuria, frequency, urgency, or lower abdominal pain [onset within 96 hours of study entry] without manifestations suggestive of upper urinary tract infection [UTI] or otherwise complicated UTI). Additionally, eligible participants had evidence of urinary nitrite or pyuria (>15 white blood cells/high-power field or 3+/large leukocyte esterase based on dipstick test), from a pre-treatment clean-catch midstream urine sample. A complete list of inclusion and exclusion criteria has previously been published [9].

### Study Analyses

Therapeutic, clinical, and microbiological success at TOC, overall and by subgroup, were assessed in the pooled microbiological intent-to-treat nitrofurantoin-susceptible (micro-ITT NTF-S), micro-ITT NTF-NS, and in the microbiologically evaluable at TOC (ME-TOC) NTF-S and ME-TOC NTF-NS populations. The micro-ITT population included randomized participants who received at least one dose of study treatment and had one or two baseline qualifying uropathogens (≥10^5^ CFU/mL); participants with only NTF-S qualifying uropathogens were included in the micro-ITT NTF-S population; participants with any NTF-NS (ie, resistant, intermediate, or no interpretation) qualifying uropathogens were included in the micro-ITT NTF-NS population. Susceptibility was determined according to Clinical and Laboratory Standards Institute (CLSI) guidelines [13]. Pathogens with a nitrofurantoin susceptibility result of “no interpretation” means that the CLSI has not established clinical breakpoints for that antimicrobial agent-organism combination; therefore, these organisms cannot be considered susceptible to nitrofurantoin. Participants included in the micro-ITT populations with missing outcome assessments or who received non-study systemic antibacterials through TOC were considered to have clinical/microbiological/therapeutic failure.

The ME-TOC NTF-S and ME-TOC NTF-NS populations included participants from the micro-ITT NTF-S and micro-ITT NTF-NS populations who followed important components of the study: [1] received at least 80% of planned doses as randomized and actual treatment received was the same as randomized; [2] had a quantitative urine culture at TOC and evaluable clinical scores at baseline and TOC; [3] did not receive other systemic antibacterials that could potentially confound the outcome assessments; and [4] had no other major protocol deviation that prevented evaluation of efficacy. To investigate efficacy across participant subpopulations, including those at risk of drug-resistant uropathogens and/or treatment failure, subgroups defined by the following characteristics were pre-planned: age; race; ethnicity; geographic region; uUTI recurrence history; age group and recurrence history combinations; infections due to any extended-spectrum β-lactamase positive (ESBL+) uropathogens; infections due to any fluoroquinolone-resistant (FQ-R) uropathogens; body mass index; diabetes mellitus; baseline total symptom score; and baseline renal function.

### Statistical Analysis

Pooling data from EAGLE-2 and EAGLE-3 was appropriate as the trials shared identical study designs for study population, efficacy endpoints, and active comparator. Hypothesis testing was performed at the study level for both EAGLE-2 and EAGLE-3 to provide statistical analysis from two pivotal trials to support regulatory approval [12]. Pooled efficacy analyses were not subject to hypothesis testing; therefore, no statistical comparisons were made, and pooled results were descriptive (not multiplicity adjusted). Between-treatment differences (95% confidence interval [CI]) in success rates were calculated using the Miettinen-Nurminen (score) method, adjusting for all combinations of age group (≤50 years/>50 years), history of uUTI recurrence (recurrent/non-recurrent), and study (in the overall analysis), or adjusting for study only (in all subgroup analyses) due to smaller sample sizes in participant subgroups. Treatment differences were only calculated for subgroups with ≥10 participants across both treatment groups.

## RESULTS

### Baseline Demographics and Clinical Characteristics

Collectively, the two trials enrolled 3136 adolescent and adult female participants. Participants were predominantly enrolled in North America (61%, 1918/3136) and Europe (34%, 1071/3136), with the US as the highest enrolling country (55%, 1730/3136). Overall, 45% (1421/3136) of all randomized participants had a culture-confirmed uUTI with a uropathogen (≥10^5^ CFU/mL) qualifying for inclusion in the pooled micro-ITT population. Demographic and baseline clinical characteristics of the pooled micro-ITT NTF-S (N = 1201) and micro-ITT NTF-NS (N = 220) populations are presented in Table 1. The number of participants in each population by study is shown in Supplementary Table 1. Characteristics were broadly similar between analysis populations, although a lower proportion of participants in the micro-ITT NTF-S versus the micro-ITT NTF-NS population were of Hispanic/LatinX ethnicity (29% vs 42%, respectively) and were from North America (58% vs 70%), comprising mostly US participants (52% vs 65%). A higher proportion of participants in the micro-ITT NTF-S versus the micro-ITT NTF-NS population were from Europe (38% vs 27%) and had a history of recurrent uUTI (40% vs 34%). In both populations, baseline clinical characteristics were generally similar between treatment arms. The percentage of participants with a NTF-NS uropathogen by baseline characteristic is presented in Supplementary Table 2. The most common qualifying uropathogens at baseline were *Escherichia coli* (90%) in the micro-ITT NTF-S population, and *Klebsiella pneumoniae* (31%), *Proteus mirabilis* (26%), and *Escherichia coli* (24%) in the micro-ITT NTF-NS population (Table 2).

**Table 1.** Demographics and Other Baseline Clinical Characteristics of the Pooled Micro-ITT NTF-S and NTF-NS Populations

	Micro-ITT NTF-S Population			Micro-ITT NTF-NS^c^ Population		
	Gepotidacin (N = 628)	Nitrofurantoin (N = 573)	Total (N = 1201)	Gepotidacin (N = 104)	Nitrofurantoin (N = 116)	Total (N = 220)
Age, years	50.8 (18.1)	52.2 (17.8)	51.5 (18.0)	48.9 (17.1)	52.1 (16.4)	50.6 (16.8)
Age group						
<18	1 (<1)	4 (<1)	5 (<1)	1 (<1)	0(0)	1 (<1)
≥18–50	292 (46)	251 (44)	543 (45)	55 (53)	53 (46)	108 (49)
>50	335 (53)	318 (55)	653 (54)	48 (46)	63 (54)	111 (50)
Race	n = 627	n = 573	n = 1200			
American Indian/Alaskan Native	27 (4)	31 (5)	58 (5)	3 (3)	5 (4)	8 (4)
Asian	25 (4)	29 (5)	54 (5)	4 (4)	5 (4)	9 (4)
Black/African American	38 (6)	29 (5)	67 (6)	10 (10)	8 (7)	18 (8)
White	530 (85)	480 (84)	1010 (84)	86 (83)	98 (84)	184 (84)
Native Hawaiian or other Pacific Islanders	1 (<1)	0 (0)	1 (<1)	0 (0)	0 (0)	0 (0)
Multiple	6 (<1)	4 (<1)	10 (<1)	1 (<1)	0 (0)	1 (<1)
Ethnicity						
Hispanic/LatinX	193 (31)	156 (27)	349 (29)	46 (44)	46 (40)	92 (42)
Not Hispanic/LatinX	435 (69)	417 (73)	852 (71)	58 (56)	70 (60)	128 (58)
Geographic region						
North America	358 (57)	341 (60)	699 (58)	74 (71)	79 (68)	153 (70)
United States	323 (51)	307 (54)	630 (52)	70 (67)	74 (64)	144 (65)
Asia-Pacific	19 (3)	22 (4)	41 (3)	3 (3)	5 (4)	8 (4)
Europe	251 (40)	210 (37)	461 (38)	27 (26)	32 (28)	59 (27)
BMI, kg/m^2^	27.2 (5.0)	26.9 (5.0)	27.0 (5.0)	27.3 (5.3)	27.9 (4.8)	27.6 (5.0)
BMI group						
<25	223 (36)	217 (38)	440 (37)	34 (33)	33 (28)	67 (30)
25 to <30	233 (37)	200 (35)	433 (36)	38 (37)	44 (38)	82 (37)
≥30	172 (27)	156 (27)	328 (27)	32 (31)	39 (34)	71 (32)
History of diabetes mellitus						
Yes	92 (15)	92 (16)	184 (15)	8 (8)	22 (19)	30 (14)
No	536 (85)	481 (84)	1017 (85)	96 (92)	94 (81)	190 (86)
Renal function, CrCL^a^ (mL/min)	n = 613	n = 559	n = 1172	n = 104	n = 113	n = 217
Normal (≥90)	464 (76)	403 (72)	867 (74)	84 (81)	87 (77)	171 (79)
Mild impairment (≥60 to <90)	116 (19)	116 (21)	232 (20)	14 (13)	22 (19)	36 (17)
Moderate impairment (≥30 to <60)	30 (5)	38 (7)	68 (6)	5 (5)	3 (3)	8 (4)
Severe impairment (<30)	3 (<1)	2 (<1)	5 (<1)	1 (<1)	1 (<1)	2 (<1)
Baseline total symptom score	7.4 (2.5)	7.2 (2.5)	7.3 (2.5)	7.3 (2.4)	7.6 (2.2)	7.5 (2.3)
Baseline total symptom score group						
2–5 (mild)	152 (24)	144 (25)	296 (25)	22 (21)	23 (20)	45 (20)
6–8 (moderate)	264 (42)	256 (45)	520 (43)	49 (47)	54 (47)	103 (47)
9–12 (severe)	210 (34)	173 (30)	383 (32)	33 (32)	39 (34)	72 (33)
History of recurrent uUTI						
Recurrent infection^b^	262 (42)	224 (39)	486 (40)	36 (35)	39 (34)	75 (34)
Non-recurrent infection	366 (58)	349 (61)	715 (60)	68 (65)	77 (66)	145 (66)

Data are mean (*SD*) or n (%); percentages are calculated with non-missing data as the denominator.

Abbreviations: BMI, body mass index; CrCL, creatinine clearance; micro-ITT, microbiological intent-to-treat; NTF-NS, nitrofurantoin not-susceptible; NTF-S, nitrofurantoin-susceptible; SD, standard deviation; uUTI, uncomplicated urinary tract infection.

^a^Based on the Cockcroft Gault formula. Per study eligibility criteria, participants with a known CrCL <60 mL/min were excluded, so only those with previously unknown impairment were enrolled.

^b^Confirmed infection with ≥1 episode within the past three months, ≥2 episodes within the past six months, or ≥3 episodes within the past 12 months before study entry.

^c^Includes resistant or intermediate to nitrofurantoin, or no nitrofurantoin interpretation.

**Table 2.** Baseline Qualifying Uropathogens and Drug-resistant Phenotypes Comprising the Pooled EAGLE-2 and EAGLE-3 Micro-ITT NTF-S and Micro-ITT NTF-NS Populations

Baseline Qualifying Uropathogen^a^	Micro-ITT NTF-S Population			Micro-ITT NTF-NS^b^ Population		
	Gepotidacin (N = 628)	Nitrofurantoin (N = 573)	Total (N = 1201)	Gepotidacin (N = 104)	Nitrofurantoin (N = 116)	Total (N = 220)
Total number of qualifying uropathogens recovered, n	642	583	1225	122	139	261
Gram negative organisms, n (%)^c^						
*Escherichia coli*	573 (89)	524 (90)	1097 (90)	25 (20)	37 (27)	62 (24)
ESBL+	84 (13)	65 (11)	149 (12)	10 (8)	11 (8)	21 (8)
FQ-R	166 (26)	128 (22)	294 (24)	13 (11)	23 (17)	36 (14)
NTF-R	0	0	0	6 (5)	8 (6)	14 (5)
SXT-R	161 (25)	134 (23)	295 (24)	11 (9)	15 (11)	26 (10)
MDR	161 (25)	127 (22)	288 (24)	14 (11)	21 (15)	35 (13)
*Klebsiella pneumoniae*	15 (2)	17 (3)	32 (3)	41 (34)	41 (29)	82 (31)
ESBL+	2 (<1)	3 (<1)	5 (<1)	9 (7)	4 (3)	13 (5)
FQ-R	2 (<1)	6 (1)	8 (<1)	7 (6)	3 (2)	10 (4)
NTF-R	0	0	0	13 (11)	15 (11)	28 (11)
SXT-R	4 (<1)	3 (<1)	7 (<1)	9 (7)	10 (7)	19 (7)
MDR	3 (<1)	4 (<1)	7 (<1)	11 (9)	9 (6)	20 (8)
Other *Klebsiella* spp.^d^	7 (1)	7 (1)	14 (1)	3 (2)	4 (3)	7 (3)
*Proteus mirabilis* ^e^	0	0	0	34 (28)	33 (24)	67 (26)
ESBL+	0	0	0	2 (2)	0	2 (<1)
FQ-R	0	0	0	11 (9)	3 (2)	14 (5)
SXT-R	0	0	0	12 (10)	5 (4)	17 (7)
MDR	0	0	0	16 (13)	5 (4)	21 (8)
*Enterobacter cloacae* complex	1 (<1)	4 (<1)	5 (<1)	5 (4)	7 (5)	12 (5)
*Citrobacter freundii* complex	12 (2)	5 (<1)	17 (1)	1 (<1)	1 (<1)	2 (<1)
Other *Citrobacter* spp.^f^	4 (<1)	5 (<1)	9 (<1)	0	1 (<1)	1 (<1)
*Morganella morganii* ^e^	0	0	0	8 (7)	5 (4)	13 (5)
Other Enterobacterales spp.^g^	0	0	0	2 (2)	4 (3)	6 (2)
Gram-negative nonfermenters	0	0	0	3 (2)	6 (4)	9 (3)
Gram-positive organisms, n (%)^c^						
*Enterococcus faecalis*	14 (2)	7 (1)	21 (2)	0	0	0
*Enterococcus faecium*	1 (<1)	0	1 (<1)	0	0	0
*Staphylococcus saprophyticus*	15 (2)	14 (2)	29 (2)	0	0	0

Abbreviations: ESBL+, extended-spectrum β-lactamase positive; FQ-R, fluoroquinolone-resistant; MDR, multidrug resistant (resistant to ≥3 antibiotic classes); micro-ITT, microbiological intent-to-treat; NTF-NS, nitrofurantoin not-susceptible; NTF-R, nitrofurantoin-resistant; NTF-S, nitrofurantoin-susceptible; spp., species; SXT-R, trimethoprim-sulfamethoxazole resistant.

^a^A participant with 2 qualifying uropathogens is counted for each different uropathogen species/group detected. A participant with 2 qualifying uropathogens of the same species/group is counted twice. Participants could not have >2 qualifying uropathogens.

^b^Includes resistant or intermediate to nitrofurantoin, or no nitrofurantoin interpretation.

^c^Denominator is the total number of all uropathogens recovered.

^d^Includes *Klebsiella oxytoca/Raoultella ornithinolytica*, *Klebsiella aerogenes*, and *Klebsiella variicola*.

^e^Intrinsically NTF-R.

^f^For the NTF-S population, this includes *Citrobacter koseri* and *Citrobacter amalonaticus*. For the NTF-NS population, this includes *Citrobacter koseri* only.

^g^Includes *Serratia marcescens*, *Providencia rettgeri*, and *Serratia liquefaciens.*

### Efficacy

In the micro-ITT NTF-S population, treatment differences (in favor of gepotidacin) [95% CI] for therapeutic, clinical and microbiological success at TOC were: 9.6% [4.1, 15.2], 2.8% [−2.4, 8.1], and 10.4% [5.2, 15.6]. Corresponding treatment differences were higher in the micro-ITT NTF-NS population compared with the micro-ITT NTF-S population: 20.8% [9.2, 32.4], 10.3% [−1.4, 22.1], 20.7% [9.2, 32.3] (Table 3).

**Table 3.** Therapeutic, Clinical, and Microbiological Success at TOC Visit in the Pooled EAGLE-2 and EAGLE-3 Micro-ITT NTF-S and Micro-ITT NTF-NS Populations

	Micro-ITT NTF-S Population			Micro-ITT NTF-NS^b^ Population		
	Gepotidacin (N = 628) n (%)	Nitrofurantoin (N = 573) n (%)	Treatment difference (95% CI)^a^	Gepotidacin (N = 104) n (%)	Nitrofurantoin (N = 116) n (%)	Treatment difference (95% CI)^a^
Therapeutic success	346 (55.1)	261 (45.5)	9.6 (4.1, 15.2)	51 (49.0)	32 (27.6)	20.8 (9.2, 32.4)
Clinical success	423 (67.4)	371 (64.7)	2.8 (−2.4, 8.1)	62 (59.6)	60 (51.7)	10.3 (−1.4, 22.1)
Microbiological success	457 (72.8)	357 (62.3)	10.4 (5.2, 15.6)	81 (77.9)	62 (53.4)	20.7 (9.2, 32.3)

microbiological success data are participant-level (ie, all baseline qualifying uropathogens must be eradicated).

Abbreviations: CI, confidence interval; micro-ITT, microbiological intent-to-treat; NTF-NS, nitrofurantoin not-susceptible; NTF-S, nitrofurantoin-susceptible; TOC, test of cure; uUTI, uncomplicated urinary tract infection.

^a^Calculated using Miettinen-Nurminen (Score) method, adjusting for all combinations of age, history of uUTI recurrence, and study.

^b^Includes resistant or intermediate to nitrofurantoin, or no nitrofurantoin interpretation.

Overall, gepotidacin efficacy was generally consistent with nitrofurantoin across clinically important subgroups. In the pooled micro-ITT NTF-S population, therapeutic success rates at TOC favored gepotidacin, with the lower bound of 95% CIs for treatment difference >0, overall and across several clinically important subgroups, including: older age (age >50 years and ≥65 years), participants with history of recurrent uUTI, and those >50 years of age with a history of recurrent UTI (Figure 1*A*). While the therapeutic success rate among participants with diabetes was numerically higher with gepotidacin than nitrofurantoin, the 95% CI for the treatment difference included zero (Figure 1*A*). Differences in therapeutic success rate between treatment groups were generally driven by differences in microbiological response (Figure 1*C*). Clinical success rates were largely similar between treatments (Figure 1*B*). Similar trends were observed in the micro-ITT NTF-NS population, although wider CIs were seen with the smaller sample size in this population (Figures 2*A*–*C*).

**Figure 1.**
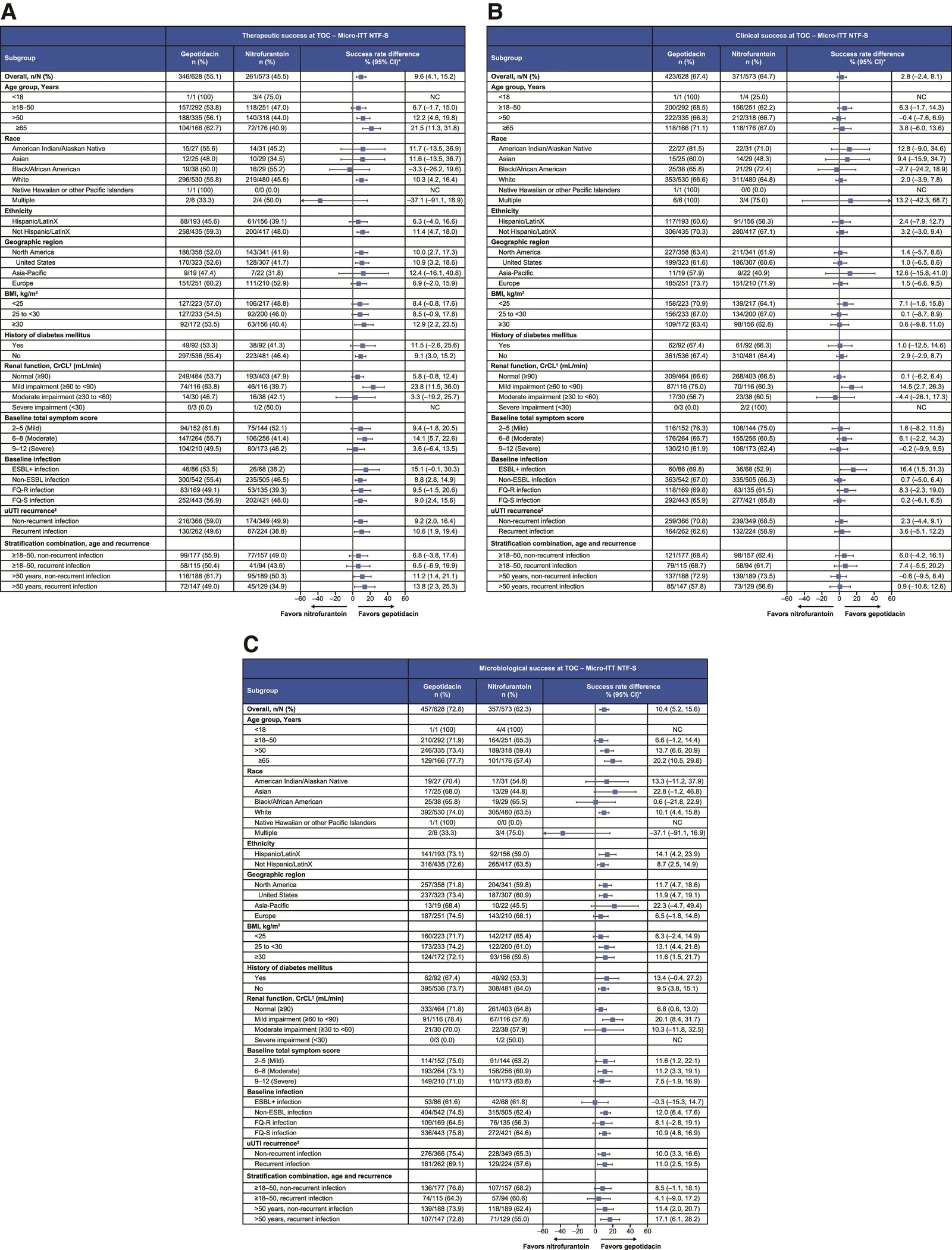
Therapeutic (*A*), clinical (*B*), and microbiological (*C*) success at TOC visit overall and by subgroup in the EAGLE-2 and EAGLE-3 pooled micro-ITT NTF-S population. *Note*: microbiological success data are participant-level (ie, all baseline qualifying uropathogens must be eradicated). Therapeutic success at TOC by baseline qualifying uropathogen in the micro-ITT NTF-S population have previously been published for the individual EAGLE-2 and EAGLE-3 studies (not for the pooled studies) [9]. *Calculated using Miettinen-Nurminen (Score) method, adjusting for all combinations of age, uUTI recurrence, and study (overall analysis), or adjusting for study only (subgroup analyses). Treatment group differences are shown for subgroups with ≥10 participants across both treatment groups. ^†^Confirmed infection with ≥1 episode within the past three months, ≥2 episodes within the past six months, or ≥3 episodes within the past 12 months before study entry. ^‡^Based on the Cockcroft-Gault formula. Per study eligibility criteria, participants with a known CrCL <60 mL/min were excluded, so only those with previously unknown impairment were enrolled. BMI, body mass index; CI, confidence interval; CrCL, creatinine clearance; ESBL+, extended spectrum β-lactamase positive; FQ-R, fluoroquinolone-resistant; FQ-S, fluoroquinolone-susceptible; micro-ITT NTF-S, microbiological intent-to-treat population whose baseline qualifying bacterial uropathogens were all susceptible to nitrofurantoin; NC, not calculated; TOC, test of cure; uUTI, uncomplicated urinary tract infection.

**Figure 2.**
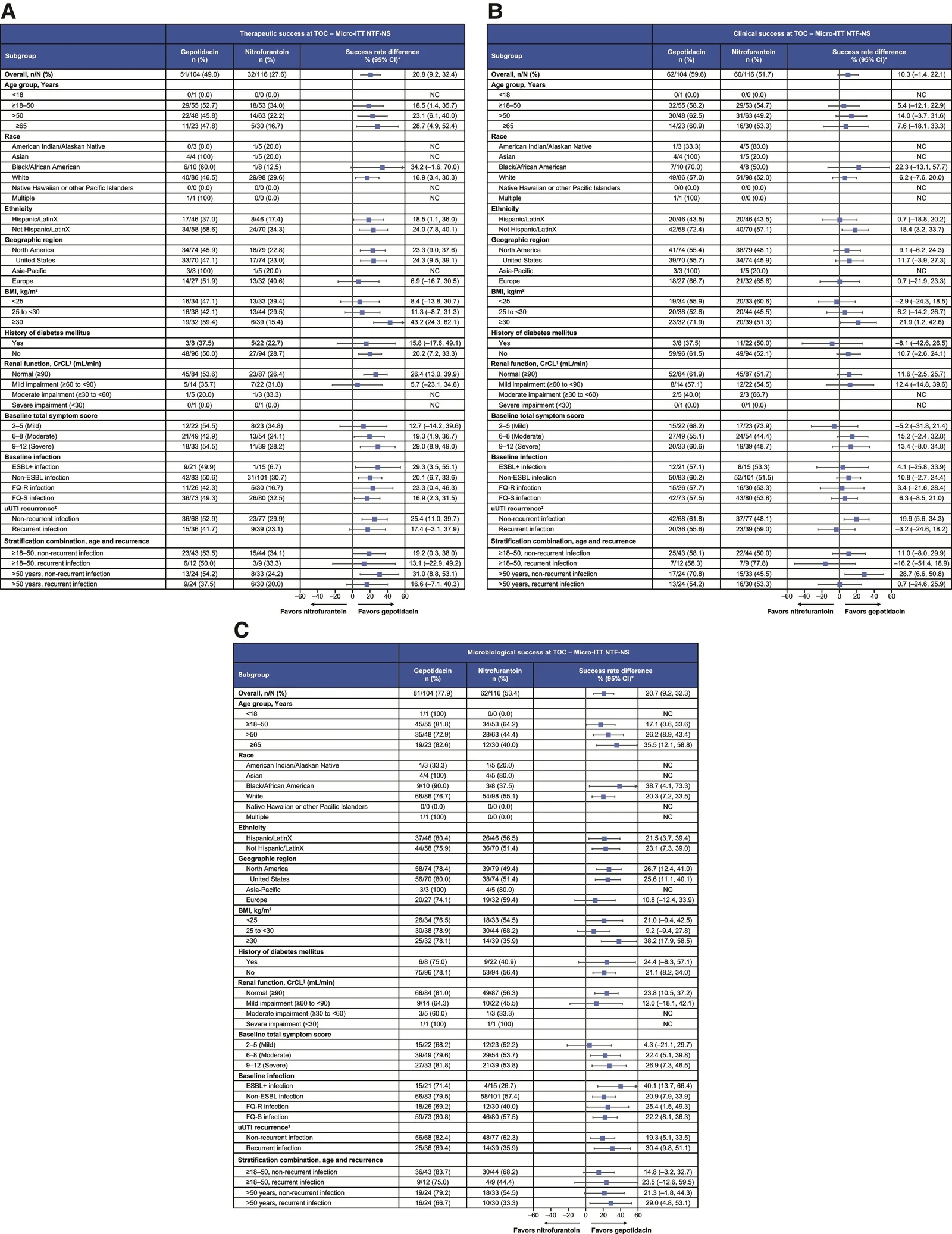
Therapeutic (*A*), clinical (*B*), and microbiological (*C*) success at TOC visit overall and by subgroup in the EAGLE-2 and EAGLE-3 pooled micro-ITT NTF-NS population. *Note*: microbiological success data are participant-level (ie, all baseline qualifying uropathogens must be eradicated). *Calculated using Miettinen-Nurminen (Score) method, adjusting for all combinations of age, uUTI recurrence, and study (overall analysis), or adjusting for study only (subgroup analyses). Treatment group differences are shown for subgroups with ≥10 participants across both treatment groups. ^†^Confirmed infection with ≥1 episode within the past three months, ≥2 episodes within the past six months, or ≥3 episodes within the past 12 months before study entry. ^‡^Based on the Cockcroft-Gault formula. Per study eligibility criteria, participants with a known CrCL <60 mL/min were excluded, so only those with previously unknown impairment were enrolled. BMI, body mass index; CI, confidence interval; CrCL, creatinine clearance; ESBL+, extended spectrum β-lactamase positive; FQ-R, fluoroquinolone-resistant; FQ-S, fluoroquinolone-susceptible; micro-ITT NTF-NS, microbiological intent-to-treat population whose baseline qualifying bacterial uropathogens were not susceptible to nitrofurantoin; NC, not calculated; TOC, test of cure; uUTI, uncomplicated urinary tract infection.

Within each treatment arm, therapeutic success rates were generally similar across participant subgroups, except as described below for older age groups, Hispanic/LatinX ethnicity, geographic region, mild renal impairment, participants with history of recurrence, and resistant uropathogens (ESBL+, FQ-R).

#### Age

In the micro-ITT NTF-S population, nitrofurantoin-treated participants in the oldest age group (≥65 years) had a lower rate of therapeutic success at TOC compared with participants aged 18–50 years (40.9% vs 47.0%) (Figure 1*A*); this pattern was also noted in the micro-ITT NTF-NS population (16.7% vs 34.0%) (Figure 2*A*). In both populations, this trend was driven by lower microbiological success rates in participants aged ≥65 years compared with those aged 18–50 years (micro-ITT NTF-S: 57.4% vs 65.3%; micro-ITT NTF-NS: 40.0% vs 64.2%; Figures 1*C* and 2*C*). This trend was not observed in the gepotidacin group where older participants (≥65 years) had a higher rate of therapeutic success at TOC compared with participants aged 18–50 years (62.7% vs 53.8%) in the micro-ITT NTF-S population (Figure 1*A*).

#### Ethnicity

In the pooled micro-ITT NTF-S population, Hispanic/LatinX participants had lower therapeutic success rates at TOC compared with non-Hispanic/LatinX participants (Figure 1*A*). This trend was noted in both treatment arms when Hispanic/LatinX participants were compared with non-Hispanic/LatinX participants (45.6% vs 59.3% in gepotidacin arm; 39.1% vs 48.0% in nitrofurantoin arm) and was driven by lower clinical success rates among Hispanic/LatinX participants (60.6% vs 70.3% in gepotidacin arm; 58.3% vs 67.1% in nitrofurantoin arm; Figure 1*B*). Across both treatment groups, microbiological success rates at TOC among Hispanic/LatinX participants were similar to non-Hispanic/LatinX participants (73.1% vs 72.6% in gepotidacin arm; 59.0% vs 63.5% in nitrofurantoin arm; Figure 1*C*). A similar trend was observed in the micro-ITT NTF-NS population (Figures 2*A*–*C*).

#### Geographic Region

In the pooled micro-ITT NTF-S population, the North America subgroup, comprising mostly participants from the US, had lower therapeutic success rates compared to Europe in both the gepotidacin arm (52.0% vs 60.2%) and the nitrofurantoin arm (41.9% vs 52.9%; Figure 1*A*). This observation was associated with differences in the clinical success rate in both the gepotidacin arm (63.4% for North America vs 73.7% for Europe) and the nitrofurantoin arm (61.9% for North America vs 71.9% for Europe) (Figure 1*B*). The microbiological success rates for gepotidacin at TOC were similar between North America and Europe (71.8% vs 74.5%), while the microbiological success rates for nitrofurantoin were lower for North America compared with Europe (59.8% vs 68.1%) (Figure 1*C*). A similar trend was observed in the micro-ITT NTF-NS population (Figures 2*A*–*C*).

#### Renal Function

In the pooled micro-ITT NTF-S population, gepotidacin-treated participants with mild renal impairment had a higher therapeutic success rate at TOC compared with participants with normal renal function (63.8% vs 53.7%); this trend did not extend to participants with moderate impairment although the small sample size (30 participants) limited interpretation (Figure 1*A*). Conversely, therapeutic success rates trended lower among nitrofurantoin-treated participants with mild and moderate renal impairment. This pattern was generally reflected in both the clinical and microbiological response endpoints (Figures 1*B* and 1*C*).

#### History of Recurrence

Within both treatment arms of the micro-ITT NTF-S population, participants with no history of recurrence had higher therapeutic, clinical, and microbiological success rates versus those with a history of recurrence (Figures 1*A*–*C*). A similar trend was observed in the micro-ITT NTF-NS population, except for clinical success in the nitrofurantoin treatment arm, which was higher in participants with a history of recurrence though this subgroup was small (Figures 2*A*–*C*).

#### Drug-resistant Uropathogens

In the micro-ITT NTF-S population, nitrofurantoin-treated participants with ESBL+ uropathogens at baseline had lower therapeutic success rates compared to participants with non-ESBL uropathogens (38.2% vs 46.5%) (Figure 1*A*); this trend was driven by a lower clinical success rate for the ESBL+ subgroup of the nitrofurantoin arm that was not observed in the gepotidacin arm (Figure 1*B*). In the gepotidacin treatment arm, microbiological success rates were higher in the non-ESBL subgroup versus the ESBL+ subgroup (74.5% and 61.6%) (Figure 1*C*). In both treatment arms, participants with FQ-R uropathogens at baseline had lower therapeutic success rates compared to participants with fluoroquinolone-susceptible (FQ-S) uropathogens (49.1% vs 56.9% in gepotidacin arm; 39.3% vs 48.0% in nitrofurantoin arm; Figure 1*A*). This trend was mostly driven by lower microbiological success rates among participants with FQ-R uropathogens (Figure 1*C*). Similar trends were observed in the micro-ITT NTF-NS population, however, differences in therapeutic success between participants with ESBL+ compared to non-ESBL uropathogens were driven by microbiological response (Figures 2*A*–*C*).

### ME-TOC NTF-S and ME-TOC NTF-NS Populations

Therapeutic, clinical, and microbiological success rates in the ME-TOC NTF-S Supplementary Figure 1) and ME-TOC NTF-NS (Supplementary Figure 2) populations were higher in both treatment arms compared, respectively, with the micro-ITT NTF-S (Figure 1) and NTF-NS (Figure 2) populations. This is as anticipated from the exclusion of participants with failure outcomes due to missing data or use of a potentially confounding non-study antibacterial received for another reason (ie, not for UTI) in the ME populations. For all three endpoints, treatment differences overall and across subgroups were generally similar between the ME-TOC NTF-S/NTF-NS and the micro-ITT NTF-S/NTF-NS populations.

## DISCUSSION

This study used pooled data from two large, contemporary clinical trials (EAGLE-2 and EAGLE-3) to explore therapeutic success rates with oral gepotidacin or nitrofurantoin treatment in subgroups of women with uUTI, including groups at higher risk for antimicrobial resistant uropathogens. Among participants with baseline uropathogens susceptible to the active control (micro-ITT NTF-S population), gepotidacin was generally associated with higher overall therapeutic and microbiological success rates compared with nitrofurantoin. As expected, among participants with NTF-NS uropathogens at baseline (representing 15% [220/1421] of the pooled micro-ITT population) treatment differences favored gepotidacin across all three efficacy endpoints (therapeutic, clinical, and microbiological response). Overall, gepotidacin was efficacious regardless of subgroup.

Lower therapeutic and clinical success rates were observed in Hispanic/LatinX participants versus non-Hispanic/LatinX participants, and among participants from North America versus Europe. These observations may be related to geographical or cultural differences in ascertaining symptom severity. Lower therapeutic, clinical, and microbiological success rates were generally observed in participants with a history of recurrent UTI versus those without, and among participants with FQ-R uropathogens compared with FQ-S. While gepotidacin is active against ESBL+ uropathogens [14], among participants treated with gepotidacin, microbiological success was lower in those with ESBL+ uropathogens compared with non-ESBL uropathogens. These observations are consistent with the heightened risk of incomplete resolution and recurrence in participants with a resistant uropathogen [15–17]. In addition, participants aged over 50 years with a history of recurrent uUTIs have been found to be at higher risk of having pathogens with antimicrobial resistance [18]. Nonetheless, therapeutic success rates were consistently similar or higher for gepotidacin compared with nitrofurantoin, underscoring the potential benefit of gepotidacin in treating participants at risk of treatment failure related to antimicrobial resistant uropathogens.

EAGLE-2 and EAGLE-3 are among the largest clinical trials ever conducted to investigate the treatment of uUTI. These trials included participants from 15 countries and substantial subpopulations of participants at higher risk of antimicrobial resistant uropathogens. In addition, the trials were among the first designed to conform with the most recent regulatory guidance for uUTI [11, 12].

Subgroup analyses in phase 3 clinical trials can provide useful insights into potential differences in treatment response; however, such analyses are subject to important limitations. The EAGLE-2/-3 trials were designed to detect overall treatment effects, not differences within subgroups, resulting in smaller sample sizes and limited statistical power for these comparisons. Although stratified randomization was performed for age category and history of recurrent UTI, apparent differences between subgroups may be confounded by baseline imbalances in prognostic factors, rather than true differences in biological effect. Furthermore, when multiple subgroups are explored, there is an increased risk of false-positive findings due to random variation. For these reasons, subgroup findings should be interpreted cautiously and viewed as hypothesis-generating.

The study entry criteria further limited enrolment of patients. For example, patients with renal impairment with known creatinine clearance <60 mL/min were excluded at study entry according to nitrofurantoin prescribing information in the US [19], even though gepotidacin is expected to be effective in patients with moderate renal impairment based on pharmacokinetic considerations [20]. Some participants who had creatinine clearance <60 mL/min which was unknown at study entry were included in the trials, but due to the exclusion criteria this was a small group and their results are not generalizable.

## CONCLUSIONS

In analyses of pooled trial data, gepotidacin was efficacious in treating women with uUTI across subgroups at higher risk for antimicrobial resistant uropathogens or treatment failure, including participants with NTF-NS uropathogens. Key subgroups included participants ≥50 years of age, participants with history of recurrent UTI, those ≥50 years of age with history of recurrent UTI, participants with diabetes, participants with ESBL+ or FQ-R uropathogens, and participants with mild renal impairment. Gepotidacin, a recently FDA approved oral treatment for uUTI, has an overall acceptable safety and tolerability profile [9], offering potential benefits to key patient subgroups.

## Supplementary Material

ciag408_Supplementary_Data

## Supplementary Data

Supplementary materials are available at *Clinical Infectious Diseases* online. Consisting of data provided by the authors to benefit the reader, the posted materials are not copyedited and are the sole responsibility of the authors, so questions or comments should be addressed to the corresponding author.
