# Supplementary material for: Subgroup Analysis of Pooled Phase 3 Efficacy Data From Two Randomized Trials of Gepotidacin Versus Nitrofurantoin in Uncomplicated Urinary Tract Infections (EAGLE-2 and EAGLE-3) Including Participants With Uropathogens Not-Susceptible to Nitrofurantoin
Source: Clin Infect Dis. 2026 Jul 8;83(4):e733–42. doi: 10.1093/cid/ciag408 (PMC13715975; doi:10.1093/cid/ciag408)
Supplement: Supplementary file 1 [file ciag408_supplementary_data.docx]

**Title:** Subgroup Analysis of Pooled Phase 3 Efficacy Data from Two Randomized Trials of Gepotidacin Versus Nitrofurantoin in Uncomplicated Urinary Tract Infections (EAGLE-2 and EAGLE-3) including Participants with Uropathogens Not-Susceptible to Nitrofurantoin

**Authors:** Amanda Sheets, PhD^1^*; Jeremy Dennison, MBChB^2^; Jennifer H. Han, MD^1^; Sarah Watts, MSc^2^; Caroline Perry, PhD^1^; Nicole E. Scangarella-Oman, MSc^1^; Deborah Butler, BS^1^; John Breton, MCM^1^; Thomas M. Hooton, MD^3^; Helen Millns, PhD^4^; Matthew Helgeson, PharmD^1^; Salim Janmohamed, MBBS^2^

**Affiliations:** ^1^GSK, Collegeville, PA, USA; ^2^GSK, London, UK; ^3^University of Miami, Miami, FL, USA; ^4^GSK, Stevenage, UK

***Corresponding author:** Amanda Sheets, GSK, 1250 S Collegeville Rd, Collegeville, PA 19426, USA. Email: amanda.j.sheets@gsk.com

**Supplementary materials**

**Supplementary Table 1.** Number of participants in the micro-ITT NTF-S and micro-ITT NTF-NS populations by study

|  | **Micro-ITT NTF-S population** | | | | **Micro-ITT NTF-NS population** | | |
| --- | --- | --- | --- | --- | --- | --- | --- |
|  | **Gepotidacin** | **Nitrofurantoin** | **Total** | **Gepotidacin** | | **Nitrofurantoin** | **Total** |
| **EAGLE-2** | 336 | 298 | 634 | 65 | | 67 | 132 |
| **EAGLE-3** | 292 | 275 | 567 | 39 | | 49 | 88 |
| **Pooled Studies** | 628 | 573 | 1201 | 104 | | 116 | 220 |

micro-ITT NTF-S, microbiological intent-to-treat nitrofurantoin susceptible population; micro-ITT NTF-NS, microbiological intent-to-treat nitrofurantoin not-susceptible population.

**Supplementary Table 2.** Percentage of participants with baseline qualifying uropathogens not-susceptible to nitrofurantoin by demographics and baseline characteristics in the pooled micro-ITT population (post hoc analysis)

|  | **Participants with a NTF-NS uropathogen, n/N (%)** |
| --- | --- |
| **Overall** | 220/1421 (15) |
| **Age, years** |  |
| 12–≤50 | 109/657 (17) |
| >50 | 111/764 (15) |
| **Race** |  |
| American Indian/Alaskan Native | 8/66 (12) |
| Asian | 9/63 (14) |
| Black/African American | 18/85 (21) |
| White | 184/1194 (15) |
| Other* | 1/12 (8) |
| **Ethnicity** |  |
| Hispanic/LatinX | 92/441 (21) |
| Not Hispanic/LatinX | 128/980 (13) |
| **Geographic region** |  |
| United States | 144/774 (19) |
| Other^†^ | 76/647 (12) |
| **BMI, kg/m^2^** |  |
| <25 | 67/507 (13) |
| 25 to <30 | 82/515 (16) |
| ≥30 | 71/399 (18) |
| **History of diabetes mellitus** |  |
| Yes | 30/214 (14) |
| No | 190/1207 (16) |
| **Renal function, CrCL**^‡^ **(mL/min)** |  |
| Normal (≥90) | 171/1038 (16) |
| Mild impairment (≥60 to <90) | 36/268 (13) |
| Moderate/severe impairment (<60) | 10/83 (12) |
| **History of recurrent uUTI** |  |
| Recurrent infection^§^ | 75/561 (13) |
| Non-recurrent infection | 145/860 (17) |

*Includes Native Hawaiian or other Pacific Islander and multiple. ^†^Includes Asia-Pacific, Europe and Mexico. ^‡^Based on the Cockcroft Gault formula. Per study eligibility criteria, participants with a known CrCL <60 mL/min were excluded, so only those with previously unknown impairment were enrolled. ^§^Confirmed infection with ≥1 episode within the past three months, ≥2 episodes within the past six months, or ≥3 episodes within the past 12 months before study entry.

BMI, body mass index; CrCL, creatinine clearance; micro-ITT, microbiological intent-to-treat population; uUTI, uncomplicated urinary tract infection.

Supplementary Figure 1. Therapeutic (A), clinical (B), and microbiological (C) success at TOC visit overall and by subgroup in the EAGLE-2 and EAGLE-3 pooled ME-TOC NTF-S population.

Note: microbiological success data are participant-level (i.e., all baseline qualifying uropathogens must be eradicated). *Calculated using Miettinen-Nurminen (Score) method, adjusting for all combinations of age, uUTI recurrence, and study (overall analysis), or adjusting for study only (subgroup analyses). Treatment group differences are shown for subgroups with ≥ 10 participants across both treatment groups. ^†^Confirmed infection with ≥1 episode within the past three months, ≥2 episodes within the past six months, or ≥3 episodes within the past 12 months before study entry. ^‡^Based on the Cockcroft-Gault formula. Per study eligibility criteria, participants with a known CrCL <60 mL/min were excluded, so only those with previously unknown impairment were enrolled.

BMI, body mass index; CI, confidence interval; CrCL, creatinine clearance; ESBL+, extended spectrum β-lactamase positive; FQ-R, fluoroquinolone-resistant; FQ-S, fluoroquinolone-susceptible; ME-TOC, microbiologically evaluable at TOC; NC, not calculated; NTF-S, nitrofurantoin-susceptible; TOC, test of cure; uUTI, uncomplicated urinary tract infection.

Supplementary Figure 2. Therapeutic (A), clinical (B), and microbiological (C) success at TOC visit overall and by subgroup in the EAGLE-2 and EAGLE-3 pooled ME-TOC NTF-NS population.

Note: microbiological success data are participant-level (i.e., all baseline qualifying uropathogens must be eradicated). *Calculated using Miettinen-Nurminen (Score) method, adjusting for all combinations of age, uUTI recurrence, and study (overall analysis), or adjusting for study only (subgroup analyses). Treatment group differences are shown for subgroups with ≥ 10 participants across both treatment groups. ^†^Confirmed infection with ≥1 episode within the past three months, ≥2 episodes within the past six months, or ≥3 episodes within the past 12 months before study entry. ^‡^Based on the Cockcroft-Gault formula. Per study eligibility criteria, participants with a known CrCL <60 mL/min were excluded, so only those with previously unknown impairment were enrolled.

BMI, body mass index; CI, confidence interval; CrCL, creatinine clearance; ESBL+, extended spectrum β-lactamase positive; FQ-R, fluoroquinolone-resistant; FQ-S, fluoroquinolone-susceptible; ME-TOC, microbiologically evaluable at TOC; NC, not calculated; NTF-NS, nitrofurantoin not-susceptible; TOC, test of cure; uUTI, uncomplicated urinary tract infection.
